# Research for development and the role of ‘grey literature’ in southern African research production

**DOI:** 10.3332/ecancer.2013.ed22

**Published:** 2013-06-12

**Authors:** Eve Gray

**Affiliations:** Centre for Educational Technology, University of Cape Town, South Africa

Open Access scholarly publishing emerged as a global movement early in this century, offering promise to developing countries not only for better access to knowledge from the global North but also visibility for research produced in the global South. Aware of the potential power of the digital knowledge economy and emerging from a difficult period in African higher education, policymakers had aspirations for a renewed African research system. Governments saw a dual promise of, on the one hand increasing their research prestige and, on the other, harnessing research production in the knowledge economy to help resolve crises of economic stagnation, poverty, public health and food sustainability.

Open access publishing offers the potential to deliver both of these aims, but an early focus on journal articles worked to the detriment of development-focused, use-based research. This is in contrast to a tradition in southern Africa of publishing longer–form publications, much of which would be classified as ‘grey literature’, with the aim of making research available beyond the confines of the research community.

More than a decade down the line, developing country research remains almost invisible at home and on the global map. The African continent is estimated to produce between 0.7% and 1% of global research publication, most of it from South Africa. Yet Africa comprises 15% of the world population. There are often sharp criticisms from government of what is seen as the failure of the research system to address urgent development priorities.

This is the result of a disjuncture between the vision set out in national research policies in Africa and the metrics used to measure performance. Journal articles are prioritized in promotion and reward systems as a way of delivering prestige and competitiveness, a narrowing of vision that consigns most of the research outputs actually produced to oblivion. There is a strong preference for publication in the Thomson Reuters journal indexes (ISI) and this is used as a proxy for research productivity in evaluations of African higher education performance.

What is not well known is that the ISI, a commercially-owned indexing system, owned by one company, has established a hierarchy of knowledge in which research from the global North is ‘mainstream’ or ‘international’ and research from the developing world as of ‘local’ interest only and therefore lacking sufficient impact for inclusion in the system unless it addresses issues that are of specific interest to Northern readers [1].

The impact of this environment is made clear by the editor of the Lancet, Richard Horton, quoted in THES in 2010 as saying that he was constrained from publishing African authors in the Lancet, because this might reduce citations from his main readership, which wants articles on randomized clinical trials in the global North. “The incentive for me is to cut off completely parts of the world that have the biggest health challenges … citations create a racist culture in journals’ decision-making and embody a system that is only about us (in the developed world).”[2]

Insight into the range of other outputs that are being produced was provided when the Carnegie 3 programme on Strategies to Overcome Poverty and Inequality launched with a conference at the University of Cape Town in late 2012 [3]. The explicit aim was to ‘seek to focus attention on understanding the lived experiences of inequality and the causes and dimensions of persistent inequality’ with the aim of finding ways of significantly reducing poverty through policy and action.

Over 300 papers were submitted and over 200 organisations participated, from universities and research councils, research groupings, NGOs, faith-based organisations, trade unions and government departments. A glance at the conference programme shows that a lot of research is being produced by a variety of individuals and organisations. The conference sought to draw on descriptive, analytical and policy research, moving towards implementation built upon demonstrations from communities, business, NGOs and trade unions. This kind of work combines both basic and applied research and recognizes knowledge that comes from beyond academe. The publications that emerge are often not formal, but fall into the category of ‘grey literature’.

The definition of grey literature, which is defined by its informality and lack of discoverability needs to be challenged in a networked scholarly environment. The latter is a problem that is easily dealt with by meta-tagging content in digital repositories, and quality assurance is being dealt with by post-publication review and in the development of quality control systems for grey literature, for example in the universities of Namibia and Botswana.

The research environment that emerges in Carnegie 3 is different to that valued in a journal culture. David Cooper in his study of use-oriented research in South Africa argues for a necessary realignment with current realities: “[A] national position needs to be articulated . . . that in the knowledge society of the third industrial revolution, issues of health, housing, transport, etc., are not independent of university research efforts” [4].

This concurs with the findings of a study of research communication at the University of Cape Town’s Opening Scholarship program [5] that identified a culture of “translational” scholarship, or what Cooper calls “use-inspired basic research,” in a number of research groupings. These produced, alongside traditional peer-reviewed articles, a range of publications, many of which were posted online on departmental websites, targeting increased impact among policymakers and communities.

Fortuitously, one of the papers delivered at the conference, by Rick de Satgé of the NGO Phuhlisani, reported on an evaluation of the availability of poverty-related information resources commissioned by the South African Presidency and the EU. The aim was to assess the need for a poverty information resource. The results of this survey, reported in De Satgé’s paper, give insight into the wide variety of research that is being produced and the range of participants involved in its production, as well as the target audiences it is reaching [6]. At the same time, de Satgé reported in an interview with the conference newspaper that a large amount of research was going to waste as academics, not rewarded for this kind of research, failed to curate it.

The list of conference papers includes a number of research organisations that have a long history of making their research production openly available. The Institute for Poverty, Land and Agrarian Studies (PLAAS) [7] at the University of the Western Cape was identified in the Phuhlisani survey as the most frequently accessed online resource. The Southern Africa Labour and Development Research Unit (SALDRU) [8] at the University of Cape Town has a 30-year history of open publication of working papers, briefing papers and policy briefs going back to an anti-apartheid commitment to rigorous research that could make a difference. SALDRU has recently worked with the Scholarly Communication in Africa Programme (SCAP) [9] in the creation of an open access repository to host its historical and current content.

The Health Economics Unit at the University of Cape Town has a departmental website that hosts reports and working papers, books and book chapters, conference papers, information sheets and policy briefs. The unit has a Communications Officer to handle production of its output, seeing to it that policy briefs, for example, are presented in the language and layout that could give them most impact. In the more than 20 years of its existence, from the very beginnings of a democratic South Africa, it has made a notable contribution to health policy development, using rigorous research to understand the changing issues facing the country and effective communications to target key audiences.

These are but a few cases from the long list of Carnegie 3 speakers and these concur with the findings of the Opening Scholarship programme [10] at the University of Cape Town, funded by the Shuttleworth Foundation, which identified a pattern of ‘translational’ research being carried out in research groupings at the university and posting outputs on the departmental website. In other words, in the most successful university in Africa in the competitive university rankings, there was a culture of development-focused research, relying on what is most often dismissed as ‘grey literature’ that explicitly seeks to gain greater impact for research among policymakers and communities. With the growth of ‘mega-journals’, the final question is how these two publishing traditions could be brought together for the sake of a more comprehensive approach to publishing the widest possible range of research.
